# A systematic review of factors that influence food store owner and manager decision making and ability or willingness to use choice architecture and marketing mix strategies to encourage healthy consumer purchases in the United States, 2005–2017

**DOI:** 10.1186/s12966-019-0767-8

**Published:** 2019-01-14

**Authors:** Bailey Houghtaling, Elena L. Serrano, Vivica I. Kraak, Samantha M. Harden, George C. Davis, Sarah A. Misyak

**Affiliations:** 1 Department of Human Nutrition, Foods, and Exercise, 337 Wallace Hall, 295 West Campus Drive, Virginia Tech, Blacksburg, Virginia, VA 24061 USA; 2 Family Nutrition Program, Department of Human Nutrition, Foods, and Exercise, Virginia Tech, Blacksburg, Virginia, USA; 3 Department of Human Nutrition, Foods, and Exercise, Virginia Tech, Blacksburg, Virginia, USA; 4 Department of Agricultural and Applied Economics, Virginia Tech, Blacksburg, Virginia, USA

**Keywords:** Food environment, Food stores, Behavioral economics, Choice architecture, Marketing mix, Healthy retail, Nutrition interventions

## Abstract

**Background:**

Altering food store environments is a promising approach to encourage healthy product purchases by consumers to improve their diet quality and health. Food store owners and managers are intermediaries to ensure that environmental changes are enacted. Despite their role as gatekeepers to implement and sustain healthy food environment changes, no systematic review has been published that examines food store owner and manager (retailer) data. Thus a review of retailer information available within the expansive United States (US) food environment literature was the purpose of this research.

**Methods:**

The PRISMA protocol was used. A search strategy, including published articles from years 1980–2017, was applied to six databases to locate relevant articles that addressed the perspective of food store retailers in the US. Data were extracted, organized, and agreed upon between two authors based on pre-designed constructs: (1) a social-ecological model to capture factors that influence retailer decision making; and (2) a marketing-mix and choice-architecture framework to examine perspectives of applied (or the prospective application of) strategies at the store-level. Study quality was assessed using quality criteria checklists for qualitative and quantitative research.

**Results:**

Thirty-one articles met inclusion criteria and most studies (*n* = 22) were qualitative and conducted in urban food stores (*n* = 23). Multiple social-ecological factors influenced retailer decision making and ability or willingness to use marketing-mix and choice-architecture strategies to improve consumers’ healthy choices to support dietary quality. These factors included: conflicting training outcomes to enhance retailers’ knowledge and skills (individual, *n* = 9); the importance of trust (interpersonal, *n* = 8); views about marketing-mix and choice-architecture strategies in the food environment (*n* = 25); consumer demand or demographics (community, *n* = 19); supplier and food store management variables (systems or sectors, *n* = 18); local and federal policy (*n* = 8); and support for community health (norms/values, *n* = 8).

**Conclusions:**

Research partnerships can support favorable business and public health outcomes to align with retailers’ business models and available resources. A participatory and translational approach to food environment research will likely maximize public health impact. Urban and rural food store retailers are important actors for future research to inform the feasibility of store retailers to apply MMCA strategies that are profitable and promote health.

**Electronic supplementary material:**

The online version of this article (10.1186/s12966-019-0767-8) contains supplementary material, which is available to authorized users.

## Introduction

The Dietary Guidelines for Americans (DGA) 2015–2020 [1] defined a healthy diet as one rich in fruits, vegetables, whole grains, lean and plant based proteins, and low-fat dairy. By these standards, dietary behaviors in the United States (US) are overwhelmingly characterized as poor [2], and foods and beverages high in saturated fats, added sugars, and sodium are commonly overconsumed [1]. The US food environment is a major influence on the dietary behaviors of consumers that increases their risk for obesity [3–6]. Several reviews of food environment research have assessed one or more strategies to improve the dietary behaviors of consumers at the point of choice in food stores [7–18]. However, no systematic review has been published to investigate the factors that influence US food store owners and managers to promote healthy food environments for consumers.

This is a notable gap as store owners and managers are ‘knowledge brokers’ [19] who could implement research-based strategies in food stores to promote population health. A popular approach to intervening in food environments is through the use of voluntary strategies to manipulate food and beverage properties and placements [20] to favor healthier products [14–18, 21–23]. For example, a number of marketing-mix and choice-architecture (MMCA) strategies [20] could be used in the food store setting to reduce the cognitive effort for US consumers to purchase DGA-aligned foods and beverages [21, 22, 24]. These behavioral economic approaches have been demonstrated effective [14–18] and base on the ideology of ‘libertarian paternalism,’ or strategies that favor human biases without restricting choice [22].

However, the main focus of this literature has been on consumer responses to MMCA use. For example, the impact of applying floor arrows [25] and altering the products available within checkout lanes on the dietary quality of consumer food and beverage purchases [26]. Nutrition interventions that apply MMCA strategies in US food stores may not be widely feasible or easily sustained from a management perspective, due to the potential for high costs and a negative impact on store revenue [24]. Food store owners and managers are critical gatekeepers to food store interventions as they are responsible for implementing and sustaining any number of MMCA strategies that aim to improve consumers’ dietary quality [27].

This research fills a notable gap by examining US food store owner and manager perspectives that are available within the expansive food store environment literature regarding factors that influence decision making and use of MMCA strategies in food stores. This research can help to inform consumer-oriented public health nutrition strategies in food stores that are economically viable for US food store businesses to implement and sustain.

## Methods

The research question used to guide the research was: *What are the influential factors that affect US food store owner and manager decision making and ability or willingness to apply marketing-mix and choice-architecture strategies to encourage healthy consumer food and beverage purchases among consumers?*

The Preferred Reporting Items for Systematic Reviews and Meta-Analysis (PRISMA) statement [28] was used to guide this systematic literature review (that is registered with PROSPERO, CRD42016042170). All of the co-investigators are professionals with a variety of expertise within the broad field of public health, including food and nutrition policy, community food systems and food environments, applied economics, and dissemination and implementation science.

### Search strategy

**Fig. 1:**
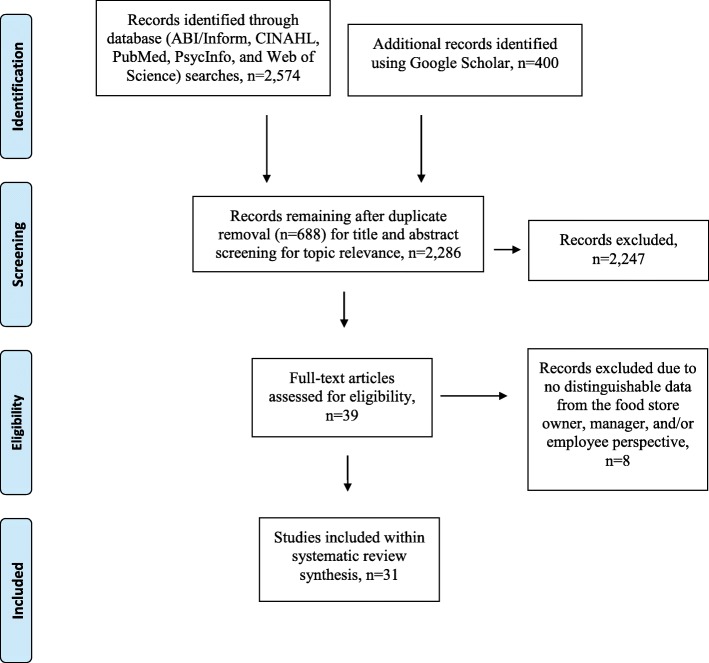
Search Protocol and Process Using the Preferred Reporting Items for Systematic Reviews and Meta-Analysis Guidelines. This figure portrays the PRISMA diagram with regard to the search parameters and outcomes of US food store owner and manager information

Five electronic databases (see Fig. 1) and Google Scholar were used to search for relevant literature published from January 1, 1980 to December 31, 2017. The year 1980 was selected due to the increased growth in overweight and obesity in US population at this time [29, 30].

Key search terms were constructed around four concepts. These concepts are detailed along with a complete list of key terms per category (displayed within respective parenthesis): (1) population (*manager(s)*, *managers*, owner*, supervisor*, *CEO*, *owner manager(s)*)*;* (2) setting (*food environment*, *store(s)*, *retail*, *food store(s)*, *corner store*, *healthy store*, *bodega*, *grocery*, supermarket*, *checkout aisle(s)*, *small food store*, *store-based*, *convenience store*); (3) nutrition (*healthy food(s)*, *nutritious option(s)*, *dietary choice(s)*, *healthy choice(s)*, *fruit(s)*, vegetable*, *whole grain(s)*, *low fat dairy*, *healthy snack(s)*, *healthy diet(s)*, *consumption*, beverage*), and; (4) MMCA strategies (*nudge framework*, *healthy nudge(s)*, *store ambience*, *store atmosphere*, *private label brand*, *portion, price(s)*, *pricing*, *cost*, *sales*, *purchase*, *food marketing*, *food promotion*, *food label*, advertis*, *product placement*, *business practices*, *product display*, *product sign*, *product signs*, *product signage*, *nutrition profile*, *nutrient profile*, *food access*, *food proximity*, *health promotion*). The key terms noted with (s) were applied in both singular and plural form.

The search protocol was constructed alongside a research librarian. Key word application differed slightly depending on the database. The complete search parameters are available upon author request.

### Inclusion and exclusion criteria

If an article was original research, peer-reviewed, published in English, within the US food store setting, and reported data from US food store owners or managers (retailers used as the terminology henceforth) it was included in the review of literature. Authors chose to limit research to the US for two reasons: (1) the existence of federal nutrition assistance programs in the US that impact local food store environments, and; (2) evidence of cross-country differences in local food environments [31].

‘Food store’ was defined broadly to include any retail location where household food and beverage purchases are made, excluding farm stands or markets. The food store setting of included research was described using the categories grocery or supermarket, drug, mass merchandiser, supercenter, convenience, dollar, club, or other (specialty/small food/corner) [32].

### Study selection, data extraction, and analysis

An EndNote database was used to capture the systematic search and to organize articles that met the criteria for data extraction (Fig. 1). Duplicate sources were removed and two authors reviewed the remaining titles and abstracts for study relevance and full text review. References of full text review articles were scanned for additional relevant research, however, no new articles were identified using this method. See Fig. 1.

Articles were excluded during full text review primarily because they did not include results from the retailer perspective in the food store setting [27, 33–37]. Two articles were excluded because food store retailers’ perspectives were collated with other stakeholder opinions [38, 39], making it impossible to discern retailer-specific data from other stakeholder insights. Also, although the search strategy was not designed to source food store employee research, this population was determined by authors to be extensions of management and therefore eligible for review inclusion.

All extracted data were compared among co-authors to ensure accuracy and to resolve discrepancies. Three authors collected pre-determined outcomes data aligned with the Cochrane Collaboration’s Tool for Assessing Risk of Bias [40]. This information is available within data tables that are referenced below.

### Theoretical frameworks

All retailer data was extracted and organized within two selected frameworks. The social-ecological model was used to describe multifaceted factors (individual, interpersonal, environmental, community, systems or sectors of influence, policy, and norms/values) [41] on retailer decision making and their ability or willingness to utilize MMCA strategies to encourage healthy consumer purchases. To categorize food environment factors identified, a MMCA framework was used (place, profile, portion, pricing, promotion, healthy defaults, priming or prompting, and proximity) (see published study for category examples) [20]. Use of the MMCA framework [20] complemented the overarching social-ecological model [41] used for primary data extraction and allowed for a more specific analysis of the food environment with regard to MMCA perspectives. Data organization among the chosen frameworks was compared and agreed upon by two authors.

### Quality assessment

Two quality assessments were implemented and scoring was completed and reconciled between two authors. The Quality Criteria Checklist for Primary Research [42] was used to evaluate the quality of quantitative research. Responses were categorized as negative, neutral, or positive based on detailed ‘yes or no’ prompts (e.g., specified outcomes, bias, representativeness, sampling, withdraws, statistical analysis, practical significance, funding) [42]. For qualitative articles the Critical Appraisal Skills Programme (CASP) checklist was utilized [43]. CASP does not provide criterion for scoring articles, however authors considered the number of ‘yes’ responses out of a maximum of ten CASP questions (e.g., appropriateness of qualitative methods, researcher-participant relationship, rigor of data analysis) [43]. A ‘yes’ response of ten was the highest possible score.

## Results

Thirty-one articles met review criteria and ranged from the years 2005 to 2017. Extracted outcome results for all studies included within the systematic review of literature are available in a supplementary table. Research included in the review was in majority specific to urban (*n* = 23) [44–66] rather than rural food store environments (*n* = 7) [67–73], and one study included both urban and rural samples [74].

This review analyzes the perspectives of 788 retailers, across a range of food store formats [32] including grocery/supermarkets [44, 50, 53, 56, 58, 59, 67–69] convenience [44, 50, 51, 60, 67–69, 71, 74], club or wholesale [62, 66], dollar [60], drug [50, 60], small food/corner [44, 46, 49, 52, 54, 56, 57, 60–66, 70–73], and specialty/ethnic stores [45, 47, 48, 55] (Additional file 1: Table S1). The foods and beverages of research focus were most commonly fruits, vegetables, and DGA-aligned [1] healthier alternatives to popular consumer products (Additional file 1: Table S1).

Study quality of many of the quantitative articles (*n* = 9) were rated poorly, scoring as either negative [53, 56, 62, 63, 66, 69, 73] or neutral [45, 71]. Scores of qualitative research (*n* = 22) were in majority positive and ranged from 0 to 10. The frequency of positive CASP [43] responses were: 0 (*n* = 3) [52, 59, 67]; 1 (*n* = 2) [48, 51]; 7 (*n* = 3) [47, 54, 60]; 8 (*n* = 4) [49, 58, 61, 68]; 9 (*n* = 3) [44, 64, 70]; 10 (*n* = 7) [46, 50, 55, 57, 65, 72, 74].

**Fig. 2:**
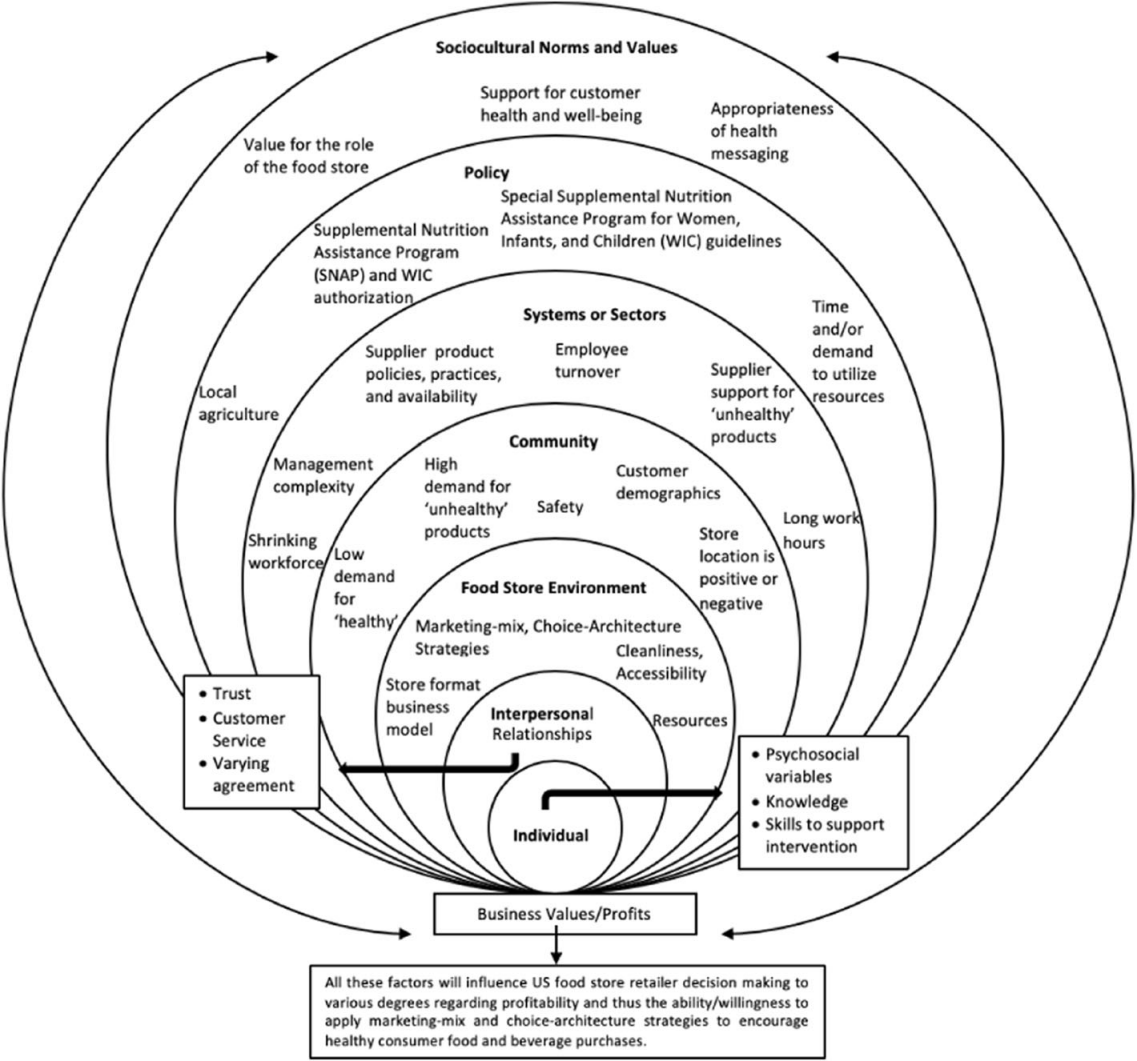
A Socioecological Visual of Influential Factors on Food Store Retailers Decision Making for Promoting Consumer Health. This figure outlines all extracted data from the US food store owner and manager perspective with regard to social-ecological factors that impact decision making

Results derived from these articles are described below with respect to social-ecological factors [41] that emerged from the data. These results are also conceptually displayed in Fig. 2. There was evidence of interrelation among social-ecological factors that influence decision making. Researchers categorized the data by best fit and these interactions are referred to throughout.

### Individual level, *n* = 9 papers

Individual-level factors that may impede or facilitate retailer ability to implement MMCA strategies (Fig. 2) were conflicting in the data analyzed. For example, interventions that aimed to support the success of healthy food objectives by targeting retailer psychosocial outcomes [62] or by providing employee trainings to enhance self-efficacy, knowledge, and customer service [53] were not always as successful as intended. Other investigations found that retailer trainings or intervention experiences were well received [45, 47], beneficial [66] and improved retailer capacity [47], self-efficacy, and knowledge to promote and stock healthy foods [56, 73]. However, ongoing retailer training/education was noted as a requirement for success [64]. Last, retailers’ perceptions of what products are ‘healthy’ did not always align with dietary guidance [50].

### Interpersonal, *n* = 8 papers

Retailer relationships with consumers, interventionists, and staff were identified as important elements that facilitated or impeded the success of food store interventions (Fig. 2). For example, some retailers perceived that establishing consumer trust influenced purchases of new products [64]. Retailers felt that good customer service was important to consumers [55] and one intervention improved retailers’ customer relations [57]. Further, enhancing retailer-interventionist trust was perceived to facilitate intervention implementation [47] and enhance the possibility for sustainability [67, 69, 73]. As one example, a study linked retailer intervention support to perceived intervention effectiveness [57].

In order to enhance trust, similar socio-cultural backgrounds of retailers and intervention/research personnel were perceived to be beneficial for establishing partnerships [57]. Finally, at times retailers and employees disagreed on appropriate intervention components or perceived consumer reactions to them [53, 55] (described more below).

### Food Store environment, *n* = 25 papers

Food store environment variables were perceived to affect the ability or willingness of retailers to implement interventions (Fig. 2). For example, the convenience store format was considered to conflict with healthy food goals due to the retailers’ described business model favoring quick-grab items rather than grocery products [64]. Retailers also described pride for clean and well-structured food store environments [72] and explained that consumers consider this important to the shopping experience [64].

Additional retailer perceptions of the food environment are organized by MMCA framework strategies [20] in Table 1. This includes retailer perspectives of applied or the prospective implementation of a variety of MMCA strategies in the food store environment to encourage healthier consumer food and beverage purchases [44, 46–50, 52, 54–60, 62–65, 67, 68, 70–72, 74]. The majority of this data is specific to the category ‘Place’ and there were often structural limitations (due to time and costs) in retailer ability to offer healthy food and beverages (Table 1). Much of this data also focused on altering food store stocking practices or ‘Profile’ and there were notable concerns for enhancing the availability of perishable products [49, 50, 65, 68]. This reservation is related to perceptions of consumer demand, described below. Also, many retailers favored ‘Promotion’ strategies [47, 48, 55–57, 64, 65, 67].

**Table 1:** Food Store Owner, Manager, and Employee Perspectives that May Impact Decision Making and Intervention Applications

First Author, Year [In-text Citation]	Retailer Perspectives
Place: Atmospheric or Structural Qualities, *n* = 15	
Andreyeva, 2011 [44]; Ayala, 2012 [46]; Caspi, 2015 [60]; Dannefer, 2012 [48]; Gravlee, 2014 [50]; Izumi, 2015 [68]; Jilcott Pitts, 2013 [74]; Kim 2017 [64]; Larson, 2013 [52]; O’Malley, 2013 [54]; Pinard, 2016 [72]; Song, 2009 [56]	Limited or lacking infrastructure (due to lack of space or equipment, time and/or cost barriers).
Baquero, 2014 [47]	Customer service and store cleanliness was improved after a nutrition intervention. The time and space required to install infrastructure is a challenge and may require interventionist assistance.
Gittelsohn, 2012 [49]	A produce display improved perceptions of the store atmosphere. The creation of a pleasant store atmosphere was of business interest.
O’Malley, 2013 [54]	Interventionist assistance would be required to implement changes.
Song, 2011 [57]	Store atmosphere was perceived to improve as a result of establishing trust.
Profile: Food Store Inventory, n = 11	
Andreyeva, 2011 [44]	Enhancing the store’s inventory is of interest to enhance product variety.
Budd, 2017 [62]	There were mixed results regarding whether retailers would sustain the stocking of promoted foods post-intervention.
Caspi, 2015 [60]	Products such as fresh fruit and low-fat milk were perceived to be low-profit items in comparison to other healthy products that were perceived as average profit items (not high profit).
D’Angelo, 2016 [71]	The following percentages of participants noted willingness to stock these items: 74%, low-fat dairy; 66%, whole grain bread; ~ 51%, three varieties each of fresh fruits and vegetables; 40%, frozen produce; ~ 40%, pre-cut fresh fruits and vegetables.
DeFosset, 2017 [63]	Variety and affordability were the most important stocking indicators and 75% considered offering healthy products a high priority.
Gittelsohn, 2012 [49]	Current store inventory informed food purchasing needs. Perishable foods were the greatest challenge with regard to predicting sales and thus ordering needs.
Gravlee, 2014 [50]	Few healthy products were currently available in stores, as indicated by participants. Product sales informed stocking needs.
Gravlee, 2014 [50]; Izumi, 2015 [68]; Mayer, 2016 [65]	Perishable products were challenging to sell before they expired.
O’Malley, 2013 [54]	There were mixed perceptions on the profitability of fresh fruits and vegetables. Sometimes the high cost of produce was considered a challenge that would require financial assistance from the intervention team.
Pinard, 2016 [72]	All except two participants expressed a willingness to stock healthier options if consumers bought them. Competition among stores in the community prompts in-store product variety. It was perceived necessary to expand inventory options that are convenient for consumers.
Pricing: Altering Costs of Food and Beverage Products, n = 3	
Kim, 2017 [64]	Small stores noted challenges to item sales due to customer expectations for continued affordability. Also, a limited variety of products available was considered a barrier to placing multiple items on sale.
Sanchez-Flack, 2016 [55]	Employees believed that tienda coupon dispensers would be an effective approach to facilitate consumer purchases, especially for produce.
Song, 2011 [57]	Believed that customers prefered product coupons rather than incentive cards.
**Pr**omotion: Increasing Consumer Demand Through Product Promotions, *n* = 8	
Baquero, 2014 [47]	Retailers reported liking the recipes and in-store food demonstrations used.
Dannefer, 2012 [48]	Participant feedback indicated that use of more posters, cooking and food demonstrations, and promotional events would be preferred.
Escaron, 2015 [67]	Perceived that deli tastings, food bundling, and promotional materials were effective in increasing consumer demand for intervention items.
Kim, 2017 [64]	Common promotional suggestions included taste testing/free samplings. Fliers were also suggested to draw consumers in.
Mayer, 2016 [65]	Some owners expressed a willingness to verbally promote healthy choices as a way to support the community.
Sanchez-Flack, 2016 [55]	Food demonstrations were discussed positively. Managers and employees believed that reusable bags for healthy foods would be an effective promotional tactic.
Song, 2009 [56]	Taste testing as a component of an intervention was perceived effective.
Song, 2011 [57]	Retailers believed the use of culturally appropriate artwork was beneficial in attracting the target intervention population. Food samples tended to be perceived as more successful than chip clip and water bottle promotions. Flyers were considered to be the least effective promotional method. Larger stores preferred posters and some perceived this method to be the most effective intervention material.
Priming or prompting: consumer cues implemented to draw attention to healthier foods and beverages, *n* = 4	
Abarca, 2005 [59]	Displays were perceived to be a possible approach to promoting sales.
Sanchez-Flack, 2016 [55]	Both managers and employees agreed on the importance of visible, well placed displays. Also, while employees tended to like the concept of using floor stickers to guide consumers with limited space, managers did not agree due to cleanliness concerns.
Song, 2009 [56]	The use of shelf labels was preferred for stores with limited space and some considered labels to be the most effective intervention method, although proper placement was important.
Wingert, 2014 [58]	Larger displays were considered to have the potential to sway consumer purchasing decisions.
Proximity: altering the location of healthy foods and beverages to reduce associated consumer effort, *n* = 5	
Baquero, 2014 [47]	Intervention infrastructure was chosen to be placed at eye-level and near a consumer check out location.
D’Angelo, 2016 [71]	The following percentages of participants noted willingness to change the location of certain items: ~ 70%, move healthy snacks and produce to checkout area; 34%, move unhealthy snacks away from checkout area.
Pinard, 2016 [72]	Perceived that placing complementary products within the same area would be a positive sales tactic. Placing aesthetically pleasing food and beverage options near the front of the store was perceived to be beneficial for sales.
Sanchez-Flack, 2016 [55]	Managers discussed the importance for effective placement of promotional reusable bags to enhance visibility.
Setala, 2011 [57]	Considered the stocking location of promoted intervention items to be an important variable.

This table includes the perspectives of United States food store owners and managers with respect to the applied or prospective application of marketing-mix and choice-architecture (MMCA) strategies to improve the dietary quality of consumer food and beverage purchases. These perspectives are within the social-ecological context of the food store environment and are influential on food store owner/manager decision making and ability or willingness to use MMCA strategies.

Less pronounced within the data are retailer perceptions of pricing strategies [55, 57, 64], the implementation of subtle consumer cues or ‘Priming or Prompting’ [55, 56, 58, 59], or alterations of placement or ‘Proximity’ [47, 55, 70–72] of consumer food and beverage options (Table 1). No extracted data fit within the MMCA categories ‘Portion’ or healthy default ‘Picks’ [20]. See Table 1.

### Community, *n* = 19 papers

Community-level variables such as perceived consumer demand, community demographic and safety factors, and the food store location were noted to drive retailer decision making and may also impact their ability or willingness to alter the food store environment (Fig. 2).

#### Consumer demand (*n* = 17)

Some retailers expressed the role of the food store as a community meeting place [49] and also indicated a preference to cater to community needs. For example, consumer taste preferences were a consideration for retailer stocking decisions [64]. Overwhelmingly consumer tastes were perceived to favor unhealthy foods and beverages [44, 54, 58, 59, 65, 72, 74] rather than healthy products [44, 48–50, 54, 57, 59, 64, 68]. As such, ceasing the sale of unhealthy items was assumed to result in lost revenue [44, 54].

Food and beverage promotions and saving potential (i.e., sales) were noted as information that influenced consumers’ purchasing decisions or product demand [55, 59, 64]. Also the importance of convenience was described, a variable that may support consumer purchases of healthy products [55, 72] even if more expensive [55]. However, retailers noted that healthy/produce products were often perceived by consumers to be more expensive to purchase [59, 63] and have less convenience attributes when compared to less healthy foods and beverages [50]. Retailers perceived consumers as amenable to an enhanced selection of foods and beverages [49] and were open to stocking products that consumers request [44, 46, 49, 50, 60, 68], so changing variables such as price/promotions [55, 59, 64] and convenience [55, 72] may help drive consumer demand (and food store offerings) toward healthier products.

#### Community safety and demographics (*n* = 8)

Some retailers considered high community crime or shoplifting rates [50, 52, 57] or drug use and prostitution [50] to strain store resources. Others perceived their consumer base to lack knowledge of healthy diets [59, 65] and to be disinterested in improving dietary behaviors to benefit health [55, 64, 65]. However, seniors and consumers with noncommunicable diseases were thought to be more willing to purchase healthy products [64]. Retailers also perceived US Department of Agriculture’s (USDA) Supplemental Nutrition Assistance Program (SNAP) participants [72] or low-income consumers in particular [57, 65] to be disinterested in purchasing healthy foods and beverages. In addition, the economic recession between years 2008–2010 (that affected all communities) was perceived to reduce the amount of healthy consumer purchases [50].

#### Food Store location (*n* = 4)

The food store location was described as beneficial or not beneficial for sales. For example, when in close proximity to certain community structures (i.e., schools) [74] or located in a dense residential area with minimal competition [52] location was considered positive. However, the rurality of a food store location was sometimes described as challenging. A shrinking consumer base in rural areas was a business concern [72]. Also, consumer demand for produce in rural areas was perceived to decrease in the summer when compared with urban locations due to rural gardening practices [74]. Partnerships with local farmers were perceived to positively impact food products stocked in some stores [73]. Rural food stores were also stated to serve as primary consumer access points that provided tailored customer services, allowing retailers to remain competitive amongst outside business competition [72].

### Systems or sectors, *n* = 18 papers

Two distinct sectors of influence emerged from the data, food store suppliers and food store management variables (Fig. 2).

#### Food Store suppliers (*n* = 15)

Retailers often noted incomplete control over the foods and beverages available in food stores. If the store was a chain or corporate location, stocking decisions were determined within upper management [50]. In addition, unhealthy products were more likely to be delivered and stocked by a supplier, while healthy options were often the retailers’ responsibility [44, 50, 57, 60, 61, 64]. Self-stocking healthy options was described as difficult to maintain due mainly to time constrains [49, 51, 64, 68]. Further, contract agreements dictated unhealthy product stocking, promotions, and placement in prime consumer areas [61, 68, 72], although were good for business despite negative potential impacts on consumer health [72]. Supplier deliveries were also linked with sale frequency [46].

Supplier product availability [64] prices [49, 54, 60, 64, 65, 68], purchasing policies (i.e., purchasing amount, package sizes, return policies) [65, 66, 68], and reliability [49, 65] impacted retailers’ product decisions. Some retailers noted that supplier recommendations and/or provided incentives influenced stocking decisions, although this was less true among others [44]. If stocking/supply barriers were present, retailers often self-supplied [46, 65] or obtained products via a combination of supplier and self-stocking methods [46, 61]. In one study, retailers self-sourced sugar sweetened beverages but were less likely to self-source or carry produce [61]. Although self-stocking was considered affordable by some, this practice also required more time [65] and some believed consumers would prefer traveling to other locations to access affordable options [74] due to supply barriers.

Store type or location was also a factor in supply decisions. For example, supplier availability and price were less important factors in stocking decisions for retailers of dollar stores and pharmacies in comparison to smaller stores [60]. Store contracts with suppliers differed by store type, i.e., lacking in small or ethic food stores in comparison to larger stores [61]. Likewise, small store retailers noted less product deliveries [61], unavailability of products, and a higher expense for healthy options in rural areas [72]. The outsourcing practices of local agricultural producers was also noted as a limitation for rural food stores who could no longer use these avenues for stocking needs [72].

#### Food Store management variables (*n* = 10)

Retailers in one study reported working long hours [65]. Others noted lacking time for processes they considered outside of the scope of their immediate job requirements [64]. Time barriers were at times hesitations to altering ‘Place’ elements in the food store environment and for self-stocking healthy products, as described above. Further, the dynamics of coordinating a business were described as costly and difficult [72]. Additional barriers included a high employee turnover rate [53] or a lack of prospective employees [72].

Business models were described as dependent on profits [60] and the convenience of operations [61]. For example, the introduction of new products was perceived as a high risk for retailers, though enhancing consumer demand was noted as a potential way to increase willingness to expand stocking selections [68]. One study noted the potential for enhanced retailer acceptance of intervention components if food store resources were not strained [67]. Further, some retailers expressed that operating within the small store context may hinder interventions due to continued low profits [65].

Implemented interventions may not translate into long-term changes of store practices [73] and also may be disruptive to store operations and components of an intervention [53]. Competition with other food stores also impacted retailer decisions and may decrease store revenue [72] and influence the ability or willingness to offer healthier consumer options [44].

### Policy, *n* = 8 papers

Various policies influenced retailer decision making and impacted store food environments and/or consumer demand (Fig. 2). One study noted that local policies disallowed retailers from utilizing nearby agriculture avenues to support healthy food stocking practices [70]. Mandated USDA Special Supplemental Nutrition Assistance Program for Women, Infants, and Children (WIC) food package changes [75] were described to widen the consumer base and positively impact profits [49] through increased consumer demand for new food and beverage requirements [44, 46]. However, fresh produce sales were perceived to increase less in comparison to other package items [46]. Due to the requirements for WIC-authorized stores to expand stocking practices to reflect package items, retailers noted that product diversity was enhanced [49].

Federal guidelines for SNAP and WIC benefit distributions were perceived to positively impact retailers through increased revenue when benefits were released to program participants [65, 72], specifically via fruit sales as noted in one study [65]. Some retailers expressed a lack of consumer demand for store SNAP or WIC authorization [74] or noted paperwork and stocking regulations as hindering to store participation [72].

### Sociocultural norms and values, *n* = 8 papers

Retailers perceived their food stores to contribute positively to their communities [52, 72] and expressed interest in supporting community needs [45, 57, 64, 65, 70, 72] and in a culturally appropriate manner [49]. Retailers engaged with the community were more responsive to store changes than retailers with less community ties [64]. Other perspectives surrounding the role of a food store in promoting consumer health included supporting families within the consumer base [52], children’s health outcomes [65, 70], and helping to mitigate high observed rates of noncommunicable diseases [65]. Retailers in one study perceived store changes to impact the health of the community, however in another study retailers worried that promoting consumer health might be considered offensive to their base [64].

## Discussion

This review of research used a social-ecological and a MMCA framework to organize and synthesize retailer perspectives available within the US food store environment literature. While the literature search was designed to retrieve research from as early as 1980, the year 2005 was the earliest publication meeting inclusion criteria. This is likely because research outcomes in these earlier years focused mainly on defining the role of food access on consumer obesity [6, 76] and designing measurement tools to distinguish ‘healthy’ versus ‘unhealthy’ nutrition environments [77].

In response to the posed research question, results indicate a multitude of factors spanning the social-ecological model influence retailer decision making and their ability or willingness to use MMCA strategies (Fig. 2). These factors are within the context of the purpose or value of a business in the US, where the outcome of interest is profit (*Profit = Revenue – Cost*) [24] (Fig. 2). Following is a discussion of key results with research, practice, and policy implications regarding the most prominent retailer themes.

It was outside the scope of this review to analyze retailer training or intervention protocol. However, the **individual-level** factors identified described food store retailers conflicting responses to trainings or interventions designed in part to enhance retailer aptitude to deliver and sustain interventions [45, 47, 53, 56, 62, 64, 66, 73]. This is a notable as “training” is perceived to be a strong implementation strategy [78] that improves high quality and sustained intervention delivery. The general guidelines for training are to be a) ongoing and b) dynamic [79]. It is inconclusive, however, as to what training should entail for US food store retailers in urban and rural areas. Only a small number of studies have reported on retailer outcomes in response to trainings or technical assistance [45, 47, 53, 56, 62, 64, 66, 73] and to the authors’ knowledge no publications fully explore retailer responses to training protocol, implementation, and fidelity for example. Future research is needed to determine pragmatic and tailored training strategies to improve food store retailer buy-in and intervention capacity.

In the food environment **interpersonal** relationships between retailers and their customers, intervention staff, and subordinates impact decision making and intervention success. The strongest shared theme was the value of trust as a mechanism to improve the success of intervention implementation and enhance the possibility for sustainability [47, 57, 67, 69, 73]. The need for trust-building is unsurprising as it is known to have a ripple effect among intervention stakeholders [19]. In this case, food store retailers are key intermediaries between researchers and consumers, staff, and subordinates. Trust between researchers/practitioners and the food store retailer is imperative to ensure that proposed interventions fit the needs and resources of the system (e.g., are not in competition with policy or profit) [80]. One strategy moving forward may be to engage in dissemination practices [81, 82] that keep retailers informed and involved throughout the entire process of intervention development. Future work should detail such efforts.

**Food store environment** factors (Fig. 2) in majority included information on the application of MMCA strategies at the store level from the retailer perspective [44, 46–50, 52, 54–60, 62–65, 67, 68, 70–72, 74]. Most of the analyzed data was focused on structural/atmospheric store properties or the types of foods and beverages stocked (Table 2). Overwhelmingly it is clear practitioners and intervention teams need to consider the potential for limited space and resources (time, money, equipment) for the design of MMCA strategies that meet store retailers ‘where they are’ currently. Also, raising consumer demand alongside any expanded food and beverage stocking is essential to prevent perishability and a loss of revenue, and has been noted previously [27].

MMCA strategies use environmental subtleties [22] to enhance consumer demand for selected products [14–16, 18]. The results presented within this review greatly add to the literature as the context (i.e., retailer perspectives, systems, sectors) of applied MMCA strategies has been under-considered in nudge research [80]. However, there is limited data on the success or uptake of applied behavioral economic strategies from the retailer perspective and more information is warranted spanning various locations and retailer/consumer cultures. Longitudinal and natural experiment designs may be useful for future MMCA research aiming to measure retailer outcomes alongside the dietary quality of consumers’ product purchases.

Additionally, this review identified retailer perspectives that at times misalign with current literature on consumer responses to MMCA strategies. A recent review of randomized control trials of food store nutrition interventions (in practice or simulated) noted that consumer coupons or vouchers were most likely to favorably nudge consumer behavior [17]. The results of this review offer very few perspectives on the feasibility or willingness of retailers to use pricing strategies in support of healthier consumer purchases [55, 57, 64]. Moving forward, a greater exploration of retailer perspectives on the use of MMCA strategies is needed to understand those strategies likely to meet both business and public health goals. In turn retailer perspectives could inform consumer investigations exploring the efficacy of MMCA strategies on healthy product purchasing to speed the translation of MMCA theory to practice.

The food store **community** also influences retailers. Overall there was a general low perceived consumer demand for DGA-aligned foods and beverages [44, 48–50, 54, 57, 59, 64, 68]. Concerns of low nutrition knowledge or interest among consumers [55, 59, 64, 65] and community crime [50, 52, 57] were also prominent. In low resource communities it may be advisable to implement retail objectives alongside community social interventions that improve consumers’ quality of life in order to impact community health in a more robust and sustainable way [83]. One example within the scope of food access is the concept of introducing a grocery business in a disparate area that has been found to enhance community economic capacity [84]. More interdisciplinary research is needed to identify similar community outcomes of food store interventions (expanding beyond dietary impact). Finally, rural food environments remain understudied [12] and require more investigation.

Supply and management realities are **systems or sectors** that impact retailers. Interestingly, retailers noted the added time or effort required to stock healthy foods and beverages in comparison to unhealthy products, which are often delivered and stocked directly by manufacturers [44, 49–51, 57, 60, 61, 64, 68]. Given the management challenges identified such as long work hours [65], high employee turnover [53], a shrinking prospective workforce [72], and slim profit margins [72], it is not surprising that the foods and beverages that are delivered and stocked *for* rather than *by* retailers are those represented in food environments. Given this, more supplier network research is warranted as conducted by Mui et al. (2015) [85], because this sector was found to considerably influence retailer decision making behaviors. Future research should also investigate the opportunity scale up and scale out [86] delivery or supply strategies that minimize the time cost for retailers to meet healthy retail objectives [87].

Both local and federal **policy** were identified as influential on retailers. Perspectives of the WIC food package changes that required authorized retailers to align the food store inventory with WIC participant allowable food and beverage purchases [75] were most represented. A review of WIC policy revisions indicated a favorable impact on consumer food environments and consumer behaviors [88]. Retailers were mainly positive regarding stocking healthy products in response to the policy change [44, 49, 88]. Perhaps this was due to ensured consumer demand [75] that impacted retailers’ favorability for in store changes, which mirrors the concept of strategic corporate social responsibility [24]. This indicates that facilitating SNAP participant purchases of healthy foods and beverages in SNAP-authorized food stores may help to overcome the barrier of no ensured demand [89] alongside a recent policy rule adjustment that aims for retailers to enhance healthy food access [90, 91].

Last, retailers’ **sociocultural norms and values** highlighted the importance placed on the health and well-being of store consumers [45, 49, 57, 64, 65, 70, 72]. While there are competing interests [24], this research captured shared, similar retailer values with public health. Framing a program as both low risk and targeted at improving the health and wellbeing of a community may be effective for building retailer-practitioner partnerships.

## Study limitations

Results are limited in transferability to other locations and contexts given the small number of studies identified, the high amount of qualitative articles, and the mainly urban settings. It is possible the search syntax was ineffective in capturing all literature relevant to review scope. The incorporation of healthy retail toolkits or gray literature was not a focus of this review. Therefore, results may have failed to provide a complete synthesis of retailer perspectives available.

In addition the varying reporting specifications or styles for qualitative research may have been a barrier for assessing study quality, although in majority ratings were positive. Many of the quantitative articles were poorly rated, although available tools do prioritize highly controlled designs. Such approaches to complex systems investigations, including retailer-focused research in the food environment setting, may be unsuitable [82].

## Conclusions

Multiple social-ecological factors impact retailer decision making and willingness or ability to support healthy food and beverage objectives in food stores. Overall, there is a dearth of retailer information available within the literature. Research approaches and intervention plans must align with retailer goals, business models, and available resources. Review results should be used to guide future investigations and research-practice partnerships that support favorable business and public health outcomes. The processes of these approaches should be rigorously documented and disseminated. More research is also needed to inform the application of numerous consumer-oriented MMCA strategies that ensure retailer profits initially and over time. Additionally, a participatory and translational approach to food environment research should be utilized to maximize public health impact.

## Additional file

Additional file 1: **Table S1.** Characteristics of original research included within systematic review of store owner, manager, and employee data (DOCX 33 kb)

## Abbreviations

CASP: Critical Appraisal Skills Programme checklist
DGA: Dietary Guidelines for Americans, 2015–2020
MMCA: Marketing-Mix and Choice-Architecture
PRISMA: Preferred Reporting Items for Systematic Reviews and Meta-Analysis
SNAP: Supplemental Nutrition Assistance Program
US: United States
USDA: United States Department of Agriculture
WIC: Special Supplemental Nutrition Assistance Program for Women, Infants, and Children
