# Supplementary material for: A systematic review of factors that influence food store owner and manager decision making and ability or willingness to use choice architecture and marketing mix strategies to encourage healthy consumer purchases in the United States, 2005–2017
Source: Int J Behav Nutr Phys Act. 2019 Jan 14;16:5. doi: 10.1186/s12966-019-0767-8 (PMC6332888; doi:10.1186/s12966-019-0767-8)
Supplement: Supplementary file 1 — Table S1. Characteristics of original research included within systematic review of store owner, manager, and employee data (DOCX 33 kb) [file 12966_2019_767_MOESM1_ESM.docx]

| Additional file 1: Table S1 Characteristics of Original Research Included within Systematic Review of Store Owner, Manager, and Employee Data | | | | | |
| --- | --- | --- | --- | --- | --- |
| Author, Year  In-text Citation | Study Design and Purpose | Store Details and Location | Participant Characteristics and Sample Size | Data Collection Procedure and Instrumentation | Foods and/or Beverages of Focus |
| Abarca et al., 2005  [59] | A cross-sectional study aiming to develop a grocery store survey specific to community indicators for nutrition | 8 Grocery stores in AZ | Managers, n=8 | Semi-structured interviews with questions about purchasing, healthy/unhealthy food demand, and barriers to purchasing healthy food | Low-fat dairy and mayonnaise, margarine, lean protein, sugar substitute and sugar-free products, olive oil, canola oil, whole wheat or grain products, 100% juice, diet soda, and sodium substitute |
| Andreyeva et al., 2011  [44] | A quasi-experiment to identify food retailors’ perceptions of healthy foods pre/post the 2009 U.S. Department of Agriculture’s (USDA) Special Supplemental Nutrition Program for Women, Infants, and Children (WIC) revisions | 68 in 2009 and 58 in 2010 WIC and non-WIC authorized convenience stores, non-chain grocery stores, and non-supermarket food marts in CT | Owners and/or managers involved in food purchases, n=68 | Pre/post survey and semi-structured interviews with questions about stocking factors, pricing/profits, WIC revisions, consumer demand, perceived barriers, and healthy food perceptions | Fresh, frozen, and canned produce, whole wheat or grain breads and cereals, low-fat dairy, tofu, soy milk, infant formula and jarred baby food |
| Ayala et al., 2012  [46] | A cross-sectional study to assess the impact of the 2009 WIC food package | 52 WIC-authorized small food stores in multiple states (MD, MN, IL, CT, LA, CA, PA, CA) | Owners and/or managers with at least 1 year of experience, n=52 | Interviews with questions about supply, products and profits, stocking factors, and perceived sales | WIC-approved foods such as fresh, frozen, and canned produce, whole grain/wheat bread, whole grain tortilla, brown rice, and low-fat dairy |
| Ayala et al., 2015  [45] | Cluster randomized controlled trial to test a multi-component intervention in small/medium tiendas to improve availability and accessibility, and consumer purchases and consumption of produce | 16 tiendas in CA | Managers at least 18 years old, work at least 20 hours/week for six months; plans to continue position for one year, decision making authority, and not employed by participating tiendas, n=16 | Interviews with questions about tiendas and manager eating habits | *Plato Total* or USDA’s MyPlate translation of the Dietary Guidelines for Americans (DGA) |
| Ayala et al., 2017  [61] | A cross-sectional qualitative investigation to understand small food store management perspectives of food and beverage supplier’s role in unhealthy food access characteristics | 72 small food stores in Baltimore MD, Durham NC, Minneapolis/St. Paul MN, and San Diego CA | 72 owners or managers of small food stores that had supplier negotiation duties that impacted stocking practices of only the store of interest, had one year or more of experience, and was 18 years of age or older | Guided interview with questions about sourcing of targeted products, agreements surrounding sourced items, placements and promotions, supplier expectations and incentivizing, and store and participant characteristics | Savory snacks, sugar sweetened beverages and snacks, confections, frozen treats, and produce |
| Baquero et al., 2014  [47] | Comprehensive process evaluation of “Vida Sana Hoy y Manana” designed to increase Latino customers' produce consumption | 4 Tiendas in NC | Managers and/or employees, n=19 | Surveys and interviews with questions about satisfaction and perceived effectiveness of intervention components | Fruits and vegetables |
| Budd et al., 2017  [62] | A randomized control trial to determine the impact of store level pricing and communication strategies on healthy product sales and prices, and store owner psychosocial indicators | 24 corner stores and 2 wholesale stores in Baltimore, MD | Owners of stores located in low income and high African American residence areas, n=24 | A pre/post intervention questionnaire with questions about demographics, sales and stocking habits of targeted foods, stocking intentions, and outcome expectations of intervention components | Low/no calorie beverages, low fat milk, wheat bread, frozen vegetables, tuna packed in water, low sugar/calorie snack alternatives, baked chips, fresh fruit |
| Caspi et al., 2015  [60] | A descriptive analysis to determine healthy food availability, stocking practices, and perceptions across types of food store establishments | Corner or small grocers, gas-marts, dollar stores, and pharmacies in MN | Owners and/or managers of both non-WIC and non-traditional food retail establishments (corner/small/dollar/gas/pharmacy stores), n=71 | Close-ended interviews with questions about stocking practices and perceptions of stocking healthy and unhealthy food products | 69 items such as fresh, frozen, and canned produce, whole grain or wheat products, 100% juice, low-fat dairy, legumes, cheese, nut butters (plain), canned fish in water, and tofu |
| D’Angelo et al., 2017  [71] | Observational and cross-sectional study to understand current store practices and willingness to change | Small food stores including grocery and convenience formats with three or fewer cash registers in NC | Independent owners and/or managers, n=55 | Demographic and scaled survey questions to assess willingness to make positive store changes | Produce, low-fat dairy, 100% whole wheat bread, healthy snacks such as fruit |
| Dannefer et al., 2012  [48] | A pre/post, mixed method evaluation of Healthy Bodegas Initiative in 2009 that aimed to increase availability and promotion of healthy foods | 55 bodegas in NY | Owners and/or managers included in intervention, n=46 | Pre/post survey assessing healthy food sales, barriers to  stocking healthy food, and intervention areas for improvement | Fresh or canned produce, low-fat dairy, no sugar added, no-low salt products, soup, healthier snack alternatives, whole grain bread, and enhanced healthfulness of convenience foods |
| DeFosset et. al., 2017  [63] | Evaluation of a food distribution program on produce access factors, member stores, and prices | 17 small stores in Los Angeles, CA | Member store representatives, n=12 | Semi-structured questionnaire assessing purchasing habits and available suppliers, and store characteristics | Fruits and vegetables |
| Escaron et al., 2015  [67] | Quasi-experiment to describe a community-academic partnership that developed and implemented "Waupaca Eating Smart (WES)," a healthy eating program in restaurants and supermarkets | 1 supermarket and 1 local convenience store chain in WI | Store operators, n=2 | Survey with scaled questions assessing healthy food specials/promotions, and response to consumer preferences | Fruits and vegetables, and deli options that include calorie limits and fruits and vegetables |
| Gittelsohn et al., 2012  [49] | Qualitative investigation to understand small grocery store owners/managers' views about 2009 WIC revisions | 52 WIC-authorized small food stores in multiple states (MD, MN, IL, CT, LA, CA, PA, CA) | Owners and/or managers in role at least one year prior to 2009 WIC revisions, n=52 | In-depth interview with questions about store operations and impact of 2009 WIC revisions | Items included in the 2009 updated WIC foods package that are based on the DGAs |
| Gravlee et al., 2014  [50] | Exploratory, mixed methods study to understand business practices, contextual factors, and food environment perceptions of store owners/managers | 2 supermarkets, 5 grocery stores, 8 convenience stores, 4 gasoline stations, and 1 pharmacy in FL | Owners and/or managers, n=20 | Semi-structured interviews to understand stocking and business practices along with a free listing exercise to elicit perceptions of healthy foods in general and those stocked in store | Not applicable |
| Izumi et al., 2015  [68] | Mixed method, convergent study to measure the nutrition environment and understand storeowner perspectives of stocking foods/beverages | 5 Grocery stores, 6 convenience  stores, and 4 gas station food marts in OR | Owners, n=6 | Semi-structured interviews with questions about snack/beverage ordering, sales, and barriers to stocking healthy snacks/beverages | The former Institute of Medicine’s (IOM) Tier 1 nutrition standards specific to healthy snacks and beverages and fresh produce |
| Jetter et al., 2010  [51] | A case study to examine impact of a pilot study aimed to increase the availability of fresh produce in a low access neighborhood | 1 convenience store in CA | Management (changes mid-intervention resulted in multiple proprietor input), n=1 | Unstructured discussions about intervention process | Fresh produce |
| Jilcott Pitts et al., 2013  [74] | A qualitative investigation to examine feasibility of increased food access with stakeholder engagement | 11 Convenience stores and food marts in NC | Rural and urban owners and/or managers, n=11 | In-depth interviews with published questions from healthy corner store website and the New Orleans Corner Store Survey | Low-fat dairy, whole wheat bread, water, baked chips, and produce |
| Kim et. al., 2017  [64] | A qualitative investigation of barriers and facilitators to stocking healthy products | 15 small stores in Baltimore, MD | Store owners, n=17 | In-depth interviews with questions focused on feasibility and effective methods for stocking healthy in addition to barrier perceptions | Products low in sugar, salt, and fat |
| Larson et al., 2013  [52] | Cross-sectional investigation aiming to increase fresh produce, low/non-fat dairy, and 100% whole wheat bread in food deserts | 5 corner stores in TN | Owners, n=5 | Semi-structured interviews with questions about strengths/challenges of food retail | Fresh produce, low-fat dairy, and 100% whole wheat bread |
| Lee et al., 2015  [53] | Evaluation of “Eat Right-Live Well!” intervention designed to increase the availability/ affordability/recognition of healthy foods to increase consumer purchases | 1 large supermarket in MD | Employees, n=63 | Employee Impact Questionnaire, survey assessing self-reported knowledge,  self-efficacy, and behavioral intent of healthy  purchasing | Healthier alternatives to popular products, as defined by the U.S. Food and Drug Administration and IOM  food selection guidelines |
| Martinez-Donate et al., 2015  [69] | Process evaluation using RE-AIM of “WES,” that aimed to improve the nutrition environment  and promote healthy eating in restaurants and food stores | 1 supermarkets and 1 local convenience store chain in WI | Operators, n=9 | Surveys that assessed intervention sustainability, business impact, and satisfaction | Fruits and vegetables, and deli options that include calorie limits and fruits and vegetables |
| Mayer et. al., 2016  [65] | A qualitative investigation to understand store roles in general and within the community in addition to challenges to operating food stores | 6 small food or corner stores in NJ and 17 in PA | Owners and/or managers involved in healthy food initiatives with an emphasis on recruiting high performance participants with regard to initiative indicators, n=23 | Interviews with questions about perceptions of health and diet, store role in the community, and reasons for participating in the healthy food initiative | Indicators for healthy foods as designated by the Healthy Corner Store Initiative |
| O’Malley et al., 2013  [54] | Mixed methods study to examine feasibility and acceptability of increased access to fresh produce | 12 corner stores in LA | Owners and/or managers, n=12 | Semi-structured interviews with questions about customers, produce, stocking characteristics, community, and interest in healthy food access collaborations | Fresh produce |
| Pinard et. al., 2016  [72] | A qualitative investigation of business and community factors in addition to the viability and ability to apply and sustain healthy food choice strategies | 15 small food stores in NE | Owners and/or managers, n=15 | Semi-structured interviews with questions about store characteristics, operations, practices, barriers based on store location, and attitudes | Not available |
| Rushakoff et al., 2017  [73] | A cross-sectional quantitative assessment to determine potential improvements to rural food stores, specifically access to and awareness of healthy foods | 10 small stores located in Cumberland Valley, KY | Owners of stores located in food deserts or food poor district areas, n=10 | A store owner survey with questions about the benefits, barriers, and impact of the food store intervention | Fresh, frozen, and canned fruits and vegetables, 100% juice, non-sugar beverages, healthy snacks, low-fat dairy, cheese, whole grain varieties, lean animal and plant-based proteins |
| Sanchez-Flack et al., 2016  [55] | Qualitative investigation to understand consumer shopping experiences | 20 Small-medium tiendas in CA | Managers and/or employees working at least 30 hours per week and employed for 6 months, n=38 | Semi-structured interviews with questions about customer base and behaviors, management, and intervention strategies | Not applicable |
| Schwendler et al., 2017  [66] | Process evaluation for the development and initiation of an intervention targeted at corner stores and wholesalers to improve healthy food access within communities | 53 corner stores and 2 wholesalers in Baltimore, MD | Owners of stores included within the intervention trial, n=29 | Process evaluation metrics that assessed intervention reach, dose, and fidelity specific to corner stores | Low/no calorie and low/no sugar beverages, low fat dairy, granola bars, 100% fruit juice, fresh, frozen and canned fruits and vegetables, seeds, low fat snacks, whole grains, low fat cooking fats, and low sodium condiments |
| Setala et al., 2011  [70] | Qualitative investigation to examine Navajo Nation farming practices to evaluate feasibility of a Farm-To-Table program to increase community produce intake | 7 small food stores in AZ | Owners, n=7 | In-depth semi-structured interviews with questions about fruits/vegetables sold and the potential to sell  local produce | Produce sourced locally |
| Song et al., 2009  [56] | Quasi-experiment that aimed to increase availability and sales of healthier food options | 13 corner stores and 4 supermarkets in MD | Owners, Korean-American, n=17 | Semi-structured interviews to gauge  storeowners’ psychosocial factors (food-related knowledge, self-efficacy for healthy  food stocking, outcome expectations, and intervention effect), and recorded physical store characteristics | Healthy alternatives to frequently reported foods and beverages consumed, aligned with the DGAs and at the same or a lower cost to purchase |
| Song et al., 2011  [57] | Exploratory investigation to understand storeowner acceptability of a healthy retail intervention | 7 corner stores in MD | Owners, Korean-American, n=7 | In-depth interviews with open-ended questions about intervention characteristics; owners were categorized by program support (weak, moderate, or strong) | Low sugar and high fiber cereal, low-fat dairy, cooking spray, reduced fat chips, low-sodium pretzels, fresh fruit, whole wheat or split top bread, diet soda, and water |
| Wingert et al., 2014  [58] | A qualitative investigation to understand low-income shoppers' perceptions of children’s influence on purchasing decisions and the supermarket environment’s role in promoting healthy options | 1 full-service supermarket in MD | Owner, n=1 | Semi-structured interview questions about product placement, availability, and potential store changes | Not applicable |
